# Successful Treatment of a Case of Aneurism by the Use of the Esmarch Bandage

**Published:** 1878-06-20

**Authors:** 


### SUCCESSFUL TREATMENT OF A CASE OF ANEURISM BY THE USE OF THE ESMARCH BANDAGE.

---

*To the Editor of the Medical Record.*

Sir—In July last Mr. R., No. —, Lexington street, Baltimore, called at my office to consult me in relation to a tumor on his right leg. He stated that he had been under treatment for two or three months, and had applied the usual remedies—iodine and other absorbents—but without any perceptible effect. The patient's age was 63; profession, a milliner; temperament lymphatic; nervous system well developed; inspiration and expiration normal; digestive organs in good condition; muscular system relaxed. The only evidence of disease was the tumor, the size of an English walnut, and the shepe of an almond, located in the region of the posterior tibial artery, in the lower third. The parts below were œdematous, but whether from the pressure of the tumor on the artery, or the obstruction of the circulation through the artery, I was unable to determine, as the tumor, owing to the thickening of its walls, betrayed no pulsation.

I then explored it with a tenotomy knife, and the resultant intermittent flow and brightly colored blood showed at once that the artery itself was involved, and the tumor necessarily an aneurismal one. Ordinarily the method of procedure would now have been to ligate the artery, but, as I did not like the appearance of the foot, owing to the extent of the effusion, I decided to apply the Esmarch bandage, beginning with the foot and carrying it above the tumor.

I did so, and after the lapse of five or ten minutes removed it and followed it with a roller-bandage, making a compress immediately over the tumor (which under the pressure of the Esmarch, had entirely collapsed and refilled slowly) with tin-foil. In this condition I left it until the following day, when I found that the foot was still œdematous, though to a less degree than before. The symptoms being favorable, and the patient professing much relief from the application, I determined to reapply the Esmarch and continue the same line of treatment.

On my next examination, three days later, I found a further very decided improvement in the patient's condition. The walls of the tumor had become consolidated, and the collateral circulation established. I now felt certain of the wisdom of the treatment, and for a third and last time applied the Esmarch. Readjusting the roller-bandage every three or four days, I found, at the end of the second week, that the œdema had entirely passed away, and in five weeks from the date of the first application the patient was able to attend to his business as usual.—*N. Y. Med. Rec.*

---

THE MEDICAL EXPERT.

Dr. J. W. Conklin publishes a paper on this subject in the *Ohio Medical and Surgical Journal*, in which he claims the importance of such testimony, and explains the disrepute into which it has fallen, by proving that experts are always called to prove the case of those who call them not to discover the truth. He advises the formation of a regular board of medical experts, as is done now in several European countries. Dr. C. proves that a physician cannot be compelled to give his opinion without sufficient remuneration, and cannot be accused of contempt for not doing so. This paper is an able one, and shows that its author has studied his subject very closely.

